# Genome-Wide Characterization and Haplotypic Variation Analysis of the *YUC* Gene Family in Foxtail Millet (*Setaria italica*)

**DOI:** 10.3390/ijms242115637

**Published:** 2023-10-27

**Authors:** Qiang Meng, Renliang Zhang, Yannan Wang, Hui Zhi, Sha Tang, Guanqing Jia, Xianmin Diao

**Affiliations:** Institute of Crop Sciences, Chinese Academy of Agricultural Sciences, Beijing 100081, China; qiangmengphd@163.com (Q.M.); zhang_renliang@163.com (R.Z.); yannanwang1999@163.com (Y.W.); zhihui@caas.cn (H.Z.); tangsha@caas.cn (S.T.)

**Keywords:** *YUC* genes, auxin, gene expression, haplotype analysis, foxtail millet

## Abstract

Panicle development and grain production in crop species are essential breeding characteristics affected by the synthesis of auxin, which is influenced by flavin monooxygenase-encoding genes such as *YUC* (*YUCCA*) family members. In this trial, fourteen *YUCs* were identified and named uniformly in foxtail millet, an ancient crop species cultivated across the world. The phylogenetic analysis revealed that the *SiYUCs* were clustered into four subgroups; protein motif and gene structure analyses suggested that the closely clustered *SiYUC* genes were relatively conserved within each subgroup; while genome mapping analysis indicated that the *SiYUC* genes were unevenly distributed on foxtail millet chromosomes and colinear with other grass species. Transcription analysis revealed that the *SiYUC* genes differed greatly in expression pattern in different tissues and contained hormonal/light/stress-responding *cis*-elements. The haplotype characterization of *SiYUC* genes indicated many superior haplotypes of *SiYUCs* correlated with higher panicle and grain weight could be favorably selected by breeding. These results will be useful for the further study of the functional characteristics of *SiYUC* genes, particularly with regard to the marker-assisted pyramiding of beneficial haplotypes in foxtail millet breeding programs.

## 1. Introduction

Grain yield formation is of great importance for the agricultural field production of crop species, which is clearly determined by panicle development that is influenced by various hormones. Plant hormones, including IAA (indole-3-acetic acid), BR (brassinosteroid), ABA (abscisic acid), and GA (gibberellin), play vital roles in crop growth regulation under variant environments. Of these, IAA was the first reported auxin associated with many biological processes in plant development [1,2] such as cell proliferation, tissue differentiation, apical dominance formation, plant architecture regulation, and determination of growing period [3,4,5,6,7,8].

The regulatory effects of auxin on plant development are mainly determined by its concentration, which is critical for maintaining plant growth under different environments [9,10,11,12]. Auxin is synthetized by tryptophan-dependent and non-tryptophan-dependent pathways in plants [3,13,14], with the tryptophan-dependent methods including four types of pathways: (i) the indole-3-acetamide (IAM) pathway; (ii) the indole-3-pyruvic acid (IPA) pathway; (iii) the tryptamine (TAM) pathway; and (iv) the indole-3-acetaldoxime (IAOX) pathway [3,13,14,15,16,17].

The *YUC* gene families encode flavin monooxygenases, which are responsible for catalyzing the conversion of TAM (tryptamine) and IPyA (indole-3-pyruvic acid) to NHT (N-hydroxyl tryptamine) and IAA (indole-3-acetic acid), respectively, during the process of auxin synthesis in plants [18,19,20,21,22,23,24]. A total of 11 *YUC* family members with functional redundancies have been identified in Arabidopsis [18,25,26,27]; transcriptional variations in *YUC* members lead to variations in IAA concentration in the root of Arabidopsis, and multiple mutations of *yuc3yuc5yuc7yuc8yuc9* severely disrupt root growth and gravity response [21,23,28]. The knockout of multiple *YUC* genes (*AtYUC1/2/4/6*) leads to narrowed leaves, while their overexpression results in leaf coiling in Arabidopsis [18,29,30]. Moreover, *AtYUC1/2/4/6* has been verified to affect leaf polar differentiation, leaf veins, and vascular bundle formation in Arabidopsis [30,31,32]. The overexpression of *AtYUC8* and *AtYUC9* in Arabidopsis result in stem regrowth [33]. *AtYUC2/6* could inhibit chlorophyll synthesis by increasing IAA concentration in Arabidopsis leaves [34]. The *YUC* gene could also impact the grain yield of crop species by influencing panicle architecture and seed development. AtYUC5 is encoded by *SUPER1* and could interact with the ERECTA receptor to regulate inflorescence architecture in Arabidopsis [26]. *Zmspi1* encodes a flavin monooxygenase; the mutation of *Zmspi1* leads to the failed initiation of BMs (branch meristems) and SPMs (spikelet pair meristems) in inflorescences and results in the generation of tassels with fewer branches and spikelets and small ears with fewer kernels [35]. *AtYUC1* and *AtYUC4* could promote floral formation, and *AtYUC10* and *AtYUC11* regulated embryo development in Arabidopsis [29,30]. *Hvyuc4* mutations lead to the failed generation of normal pollen grains due to the lack of starch and potassium in barley [36]. *OsYUC9* and *OsYUC11* were responsible for ensuring grain filling and substance accumulation [37].

Foxtail millet is an ancient crop species cultivated for grain harvesting across the globe [38]; it is being developing as a new model plant with short life cycle, high propagation coefficient, small genome size, prominent drought tolerance, and high level of photosynthesis [39,40]. In recent studies, high-quality assembled genomes and pan-genomic data have been released to facilitate genomic variation studies of foxtail millet [41,42,43,44,45]. However, the dissection of vital genomic variations associated with grain yield traits in foxtail millet have been less well investigated and many research efforts still need to be made for this important crop species.

Members of the *YUC* gene family have been identified in Arabidopsis (11, *AtYUCs*), maize (14, *ZmYUCs*), rice (14, *OsYUCs*), although variations in the *YUC* genes in foxtail millet have not been analyzed. This work elucidated genomic variation, chromosomal distribution, molecular characteristics, and expression patterns of *YUC*s of foxtail millet, as well as analyzing and identifying the superior haplotypes of several members, which was of theoretical significance for the marker-assisted pyramid breeding of this valuable crop species.

## 2. Results

### 2.1. Identification and Phylogenetic Analysis of YUCs in Foxtail Millet

To clarify the evolutionary properties of *YUC*s in foxtail millet, the *YUC* genes in foxtail millet, green foxtail, and sorghum were all identified by protein homology analysis, and a total of 14, 12, and 11 *YUCs* were detected in these species, containing the conserved FAD-binding motif (‘GxGxxG’), NADPH-binding motif (‘GxGxxG’), FMO-identifying motif (‘FxGxxxHxxxY/F’), ATG-containing motif 1[(‘Y(x)7ATGEN(x)5P’)], and ATG-containing motif 2[(‘(F/L)ATGY’)] (Figure S1).

**Figure 1:**
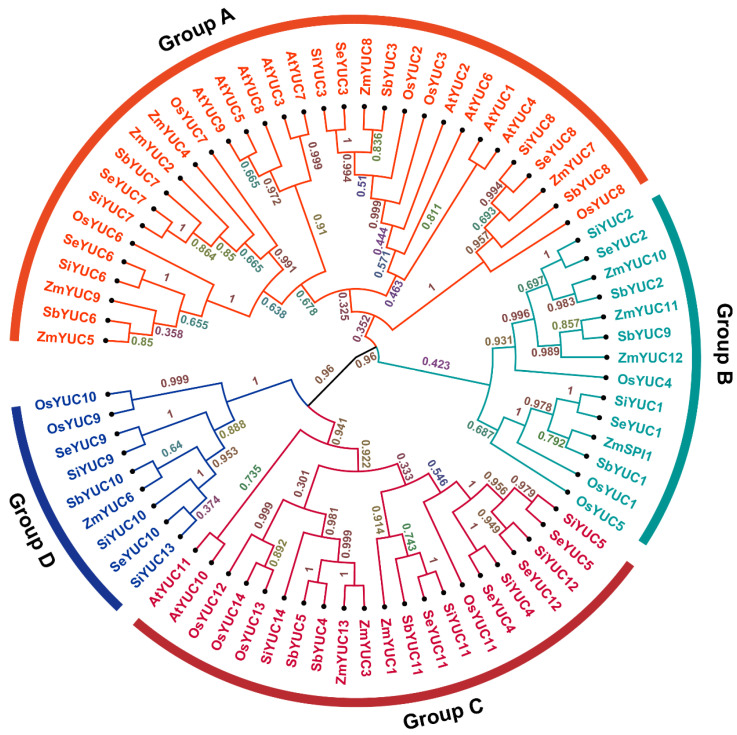
**Phylogenetic analysis of YUC proteins of foxtail millet, green foxtail, sorghum, maize, rice, and Arabidopsis.** The phylogenetic tree was constructed using the neighbor-joining method of MEGA6, with different colored branches indicating different subgroups, and branches in the same color showing that they were in one subgroup. Numbers next to branches represent the clustering confidence level. The full-length sequence of the YUC proteins was used for sequence alignment and phylogenetic analysis. The gene IDs are listed in Table S1.

The phylogenetic analysis of *YUCs* in foxtail millet, green foxtail, sorghum, maize, rice, and Arabidopsis showed that *YUC* members could be divided into four subgroups (Figure 1). *YUC* members in the same subgroup had similar numbers of introns and exons. All *YUC* members in foxtail millet were highly homologous with green foxtail, except *SiYUC13* and *SiYUC14*. This result supports the conjecture that foxtail millet was domesticated from green foxtail.

**Figure 2:**
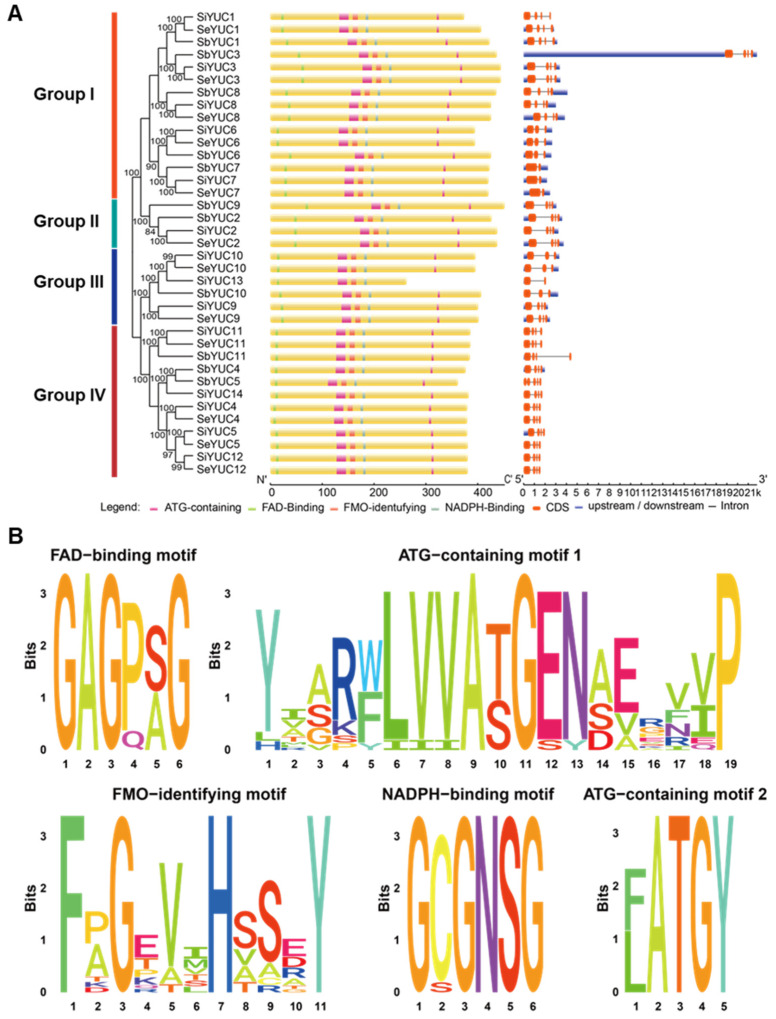
**Analysis of gene structure and conserved protein motifs of *YUC* gene family members in foxtail millet, green foxtail, and sorghum.** (**A**) Gene structure and conserved motif of *YUC* genes in foxtail millet, green foxtail, sorghum. Group I–IV and colored lines on the left indicated different clustering branches of *YUC* genes in foxtail millet (Si), green foxtail (Se) and sorghum (Sb). (**B**) Conserved motifs of proteins encoded by cereal *YUC* genes.

In general, *YUCs* of foxtail millet and green millet had similar gene structures, but there were many variations between different subgroups or between different members grouped into the same subgroup. For instance, members of subgroup I had 1~3 introns and 2~4 exons, but all the remaining subgroups contained 3 introns and 4 exons, except for *SiYUC13* (Figure 2A). All *YUC* members except *SiYUC13* in foxtail millet, green foxtail, and sorghum, contain all motifs of the *YUC* families. The FAD-binding motif and ATG containing motif 2 were identified at the N-terminus and C-terminus of the protein, respectively. The FMO-identifying motif was located in the middle of the protein, while ATG containing motif 1 and NADPH-binding motif were located before and after the FMO-identifying motif, respectively. *SiYUC13* encoded a protein without the ATG containing motif 2, which was shorter than other *YUC* members of foxtail millet (Figure 2B).

### 2.2. Structure Characterization of the YUC Proteins of Foxtail Millet

Proteins encoded by *YUCs* in foxtail millet consisted of 262~444 amino acids, with molecular weight from 28.5 to 49.4 kDa, theoretical isoelectric point from 6.48 to 9.68, instability index from 32.04 to 48.33, and hydrophilicity value from −0.181 to 0.04 (Table 1).

**Figure 3:**
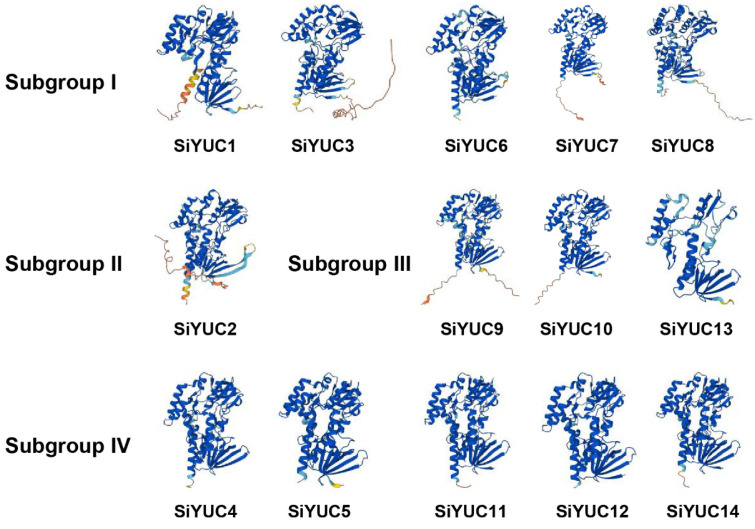
**The spatial structure predictions of SiYUC proteins.** Subgroup I–IV represented SiYUCs of different clustering branches. Details about SiYUCs protein amino acid and related prediction data links are available in Text S1.

All SiYUC proteins had similar spatial structure with conserved function except *SiYUC13* (Figure 3). Most proteins encoded by *SiYUCs* (10 out of 14) were considered stable due to the instability index being lower than 40. The SiYUC1/2/3/4/5/7/9/10/12 proteins were all hydrophilic, as hypothesized by the GRAVY value (<0) of these proteins.

### 2.3. Cis-Element Variations of YUC Members in Foxtail Millet

To investigate the possible regulatory factors of *YUC* members in foxtail millet, the *cis*-elements were identified in the promoter sequences of *YUC* genes. Almost all *YUC* promoters contained light-responsive elements, except *SiYUC11* and *SiYUC13*, which was consistent with the IAA-mediated modulation of plant phototropism. Four genes—*SiYUC3*, *SiYUC4*, *SiYUC6*, and *SiYUC10*—might be associated with drought tolerance in foxtail millet, as all of them contained an MBS (MYB binding site involved in drought-inducibility) element in their promoter regions.

The MeJA-responsiveness elements indicate that *YUC* genes might be involved in the regulation of plant defensive mechanisms, except for *SiYUC5*, *SiYUC7*, *SiYUC11*, and *SiYUC13*. Some *SiYUC* genes might be involved in the feedback regulation of IAA synthesis, as auxin response elements were identified in the promoters of *SiYUC2*, *SiYUC6*, *SiYUC9*, *SiYUC11*. TC-rich repeats and LTR (low-temperature responsiveness) elements suggest that *SiYUC6*, *SiYUC8*, and *SiYUC12* might be involved in the response to environmental stresses, such as low temperature (Table S2). The richness of regulatory elements in *YUC* promoters implied the essential regulatory roles of *YUCs* in foxtail millet growth and field production.

### 2.4. Chromosomal Distribution and Colinearity Analysis of YUCs in Foxtail Millet

**Figure 4:**
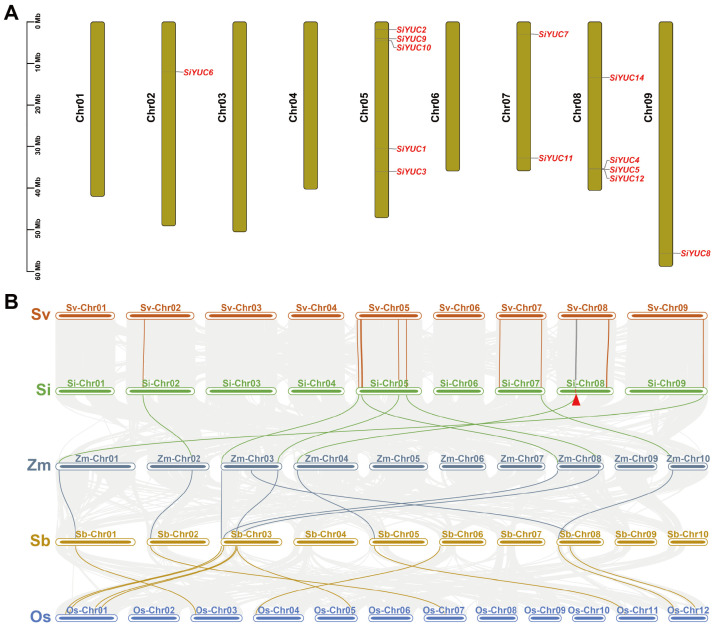
**Chromosomal distribution of *YUC* genes in foxtail millet and colinearity analysis of *YUC* genes in five grass species.** (**A**) Distribution of 13 *YUC* genes on 9 chromosomes of foxtail millet. *SiYUC13* was not shown because it was not annotated to a specific location when the genome was assembled. (**B**) Colinearity analysis of *YUC* genes in five species. The gray lines between chromosome 8 of Sv and Si indicated the colinearity of 10 genes upstream and downstream of the *SiYUC14* gene. The red triangle on chromosome 8 indicates *SiYUC14*. Sv, *Setaria viridis*, Si, *Setaria italica*, Zm, *Zea mays*, Sb, *Sorghum bicolor*, Os, *Oryza sativa*.

The *YUC* genes in foxtail millet were mainly located on chromosome 5 (with 5 members) and chromosome 8 (with 4 members). Chromosome 7 contained two *YUC* genes, and chromosome 2 and 9 contained only one *YUC* gene (Figure 4A). We simultaneously analyzed colinearity among the *YUC* genes in foxtail millet, green foxtail, maize, sorghum, and rice, and the results showed *YUC* genes from foxtail millet and green foxtail were the most colinear among all species. The *YUC* members on chromosome 5 of foxtail millet were all colinear with *YUCs* on chromosome 3 of sorghum (Figure 4B). *SiYUC13* and *SiYUC14* were specific to foxtail millet, and no homologous genes were found in green foxtail.

### 2.5. Transcriptional Profiling of YUC Members in Foxtail Millet

**Figure 5:**
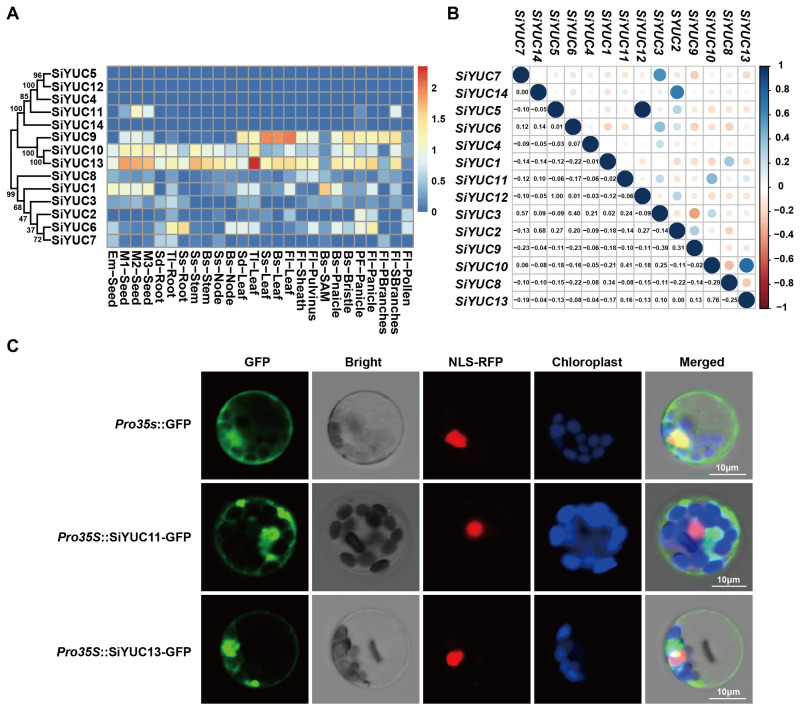
**Analysis of the transcription profile, expression correlation, and subcellular localization of the *YUC* genes in foxtail millet.** (**A**) Transcription levels of the *YUC* genes in different tissues of foxtail millet. The expression levels were defined by log_2_(FPKM+1). The tissue information for *SiYUCs* expression analysis were listed in Supplementary Table S3. (**B**) Expression correlation analysis of *YUC* genes in foxtail millet. (**C**) Subcellular localization of two YUC proteins (SiYUC11 and SiYUC13) in foxtail millet protoplast. The bar is 10 μm. NLS−RFP indicates the fusion protein of nuclear−localized signal peptide and red fluorescent protein.

To better understand the transcriptional regulation of foxtail millet growth and development controlled by the *YUC* genes, we characterized the expression pattern of the *YUC* genes in various tissues of foxtail millet at different developmental phases (Figure 5A). All *YUC* genes located on chromosome 8 had a low expression level with no tissue specificity. *SiYUC1* was mainly expressed in rapidly developing young tissues, such as young panicles, developing seeds, seedling leaves, and flag leaves, which was presumably involved in cell proliferation and tissue elongation. *SiYUC2* was mainly expressed in the leaf and panicle tissues. *SiYUC3* was expressed in all tissues except the leaves, stems, and stem nodes at the late developmental stages. *SiYUC6* had a low expression level in developing seeds, stems, stem nodes, leaves, and leaf sheaths at the shooting stage, and secondary branches of inflorescences at the flowering stage. *SiYUC7* was mainly expressed in germinating seeds, roots at the seedling stage, stem nodes at the shooting stage, and primary branches of the panicle, these results showed that *SiYUC7* might be involved in cell division or elongation. *SiYUC8* showed a tended to be constitutively expressed, except in roots, suggesting that it was mainly involved in the growth and development of the aboveground tissues of foxtail millet. *SiYUC9* was highly expressed in young leaves at the seedling stage and expanded leaves, leaf sheaths, leaf pulvini, the panicle at the flowering stage, and mature seeds. *SiYUC11* was predominantly expressed in the pre-flowering panicle, the secondary branches of the inflorescence, and mature seeds, suggesting that this gene primarily impacted the development of panicle and kernels. *SiYUC13* and *SiYUC10* had similar expression patterns and were expressed in all tissues except young panicle and anthers.

Previous studies reported that there was functional redundancy among members of the *YUC* gene family, and we hypothesized that genes with similar expression profiles might be involved in the development of the same tissue. Expressional correlation analysis between *YUC* members implied that *SiYUC3* and *SiYUC7* might be functionally redundant in the regulation of root development. *SiYUC2* and *SiYUC14* might co-regulate pre-flowering panicle development, while *SiYUC9* and *SiYUC3* showed a negative correlation in the regulation of leaves at the shooting and booting stages. *SiYUC10* and *SiYUC13* might be functionally redundant in each of the expressed tissues (Figure 5B). In addition, seed-specific expression elements were identified in the promoters of *SiYUC3* and *SiYUC5*.

The subcellular localization analysis of *SiYUC* genes revealed that they were all localized in the cytoplasm (Table 1). To verify the accuracy of this prediction, the subcellular localization of SiYUC11 and SiYUC13 were also identified by using foxtail millet protoplasts, and the results were consistent with the predicted localization (Figure 5C).

### 2.6. Haplotype Variations and Morphological Effects of YUC Members in Foxtail Millet

To investigate morphological effect of *YUC* genes on foxtail millet panicle development, we analyzed the haplotypes of the *YUC* members mainly expressed in the panicle of foxtail millet.

**Figure 6:**
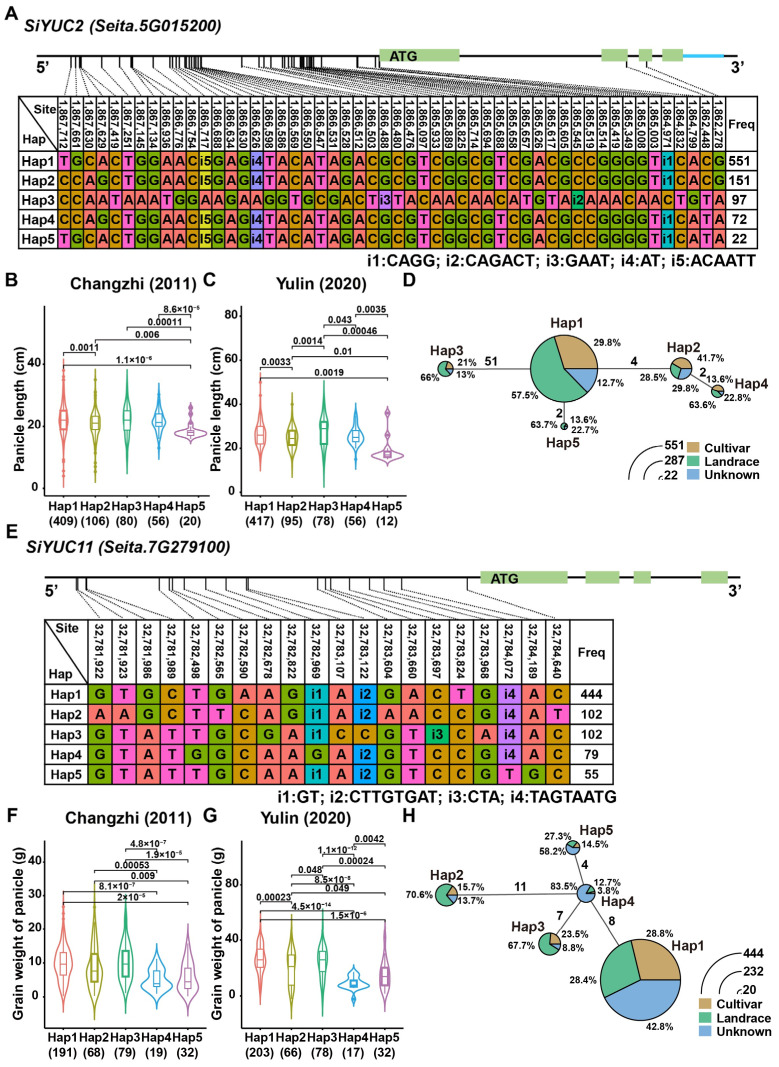
**Haplotype analysis of *SiYUC2* and *SiYUC11*.** (**A**,**E**) SNPs identified for haplotype analysis of *SiYUC2*, *SiYUC11*, respectively. Light green rectangles indicate exons, straight lines indicate introns, and SNPs in the promoter and coding sequence of the gene are shown in the upper table. The UTR region is indicated in light blue. Indel is represented by i1-i5. (**B**,**C**,**F**,**G**) The correlation analysis of haplotypes with panicle phenotypes in two different environments. The number in parentheses below the horizontal axis indicates the number of materials contained in the haplotype. Statistical significance was determined by Student’s *t* test. The material numbers used for statistical analyses are shown under each Hap. (**D**,**H**) Proportion of different foxtail millet resources among haplotypes and haplotype network analysis. Numbers above the line between haplotypes indicate the number of SNPs between them. Percentages indicate the proportion of each kind of foxtail millet resource carrying that haplotype.

A total of six haplotypes were identified in *SiYUC2* (Figure 6A); these were mainly expressed in the panicle and leaf, with Hap1 and Hap3 being the superior haplotypes with longer panicle (Figure 6B,C). The proportion of cultivars (29.8% and 21%) containing both the Hap1 and Hap3 haplotypes was lower than that of landraces (57.5% and 66%), suggesting that these two haplotypes had not been fully selected as superior haplotypes. Meanwhile, Hap2, with a relatively short panicle, constituted a large proportion of cultivars (41.7%), suggesting that this haplotype had been selected in breeding programs (Figure 6D). *SiYUC6* was mainly expressed in the panicle and roots and was categorized into six haplotypes (Figure S2A). Hap6-carrying materials, of which the cultivar (50%) constituted a higher proportion than the landrace (20%), had a thicker panicle (Figure S2B–D). However, the small number of cultivars containing this haplotype implied that Hap6 of *SiYUC6* might be beneficial as a selected target in future breeding programs (Figure S2D). The relatively higher expression of *SiYUC8* in seeds implied that it might be involved in the regulation of seed size or weight. Haplotype analysis showed that Hap1 had a higher grain weight per panicle than Hap2 (Figure S2F,G). Moreover, Hap1 of *SiYUC8* was the dominant haplotype, present at a higher proportion in cultivars (29.4%) than Hap2 (1.3%), suggesting that Hap1 might have undergone positive selection during foxtail millet breeding (Figure S2H). Similarly, a total of five haplotypes were identified in *SiYUC11*, which was specifically expressed in the seeds and the secondary branches of flowering panicles (Figure 6E). Hap1 and Hap3 were the superior haplotypes of *SiYUC11* with higher panicle grain weights (Figure 6F,G). Both Hap1 and Hap3 of *SiYUC11* were favorably carried in cultivars, which implied that they had been selected during foxtail millet breeding (Figure 6H).

## 3. Discussion

### 3.1. YUC Family Members Are Structurally Conserved and Highly Colinear in Grass Species

Members of the *YUC* gene family have been identified and functionally validated in many plants, including Arabidopsis, rice, and maize [46,47], but there is a lack of relevant research reports in foxtail millet, green foxtail, and sorghum. In this trial, we identified 14, 12, and 11 *YUC* genes in foxtail millet, green foxtail, and sorghum, respectively, based on gene homology and conserved domains. The number of *YUC* genes in foxtail millet was consistent with rice and maize [46,47], suggesting that members of this family were conserved during plant evolution. There were also differences in the number of *YUC* genes compared to Arabidopsis and sorghum, suggesting that there also some uniqueness among the species. Differences in the number of *YUC* genes between foxtail millet and green foxtail may be related to evolution or domestication. For example, *SiYUC13* and *SiYUC14* might have been generated by gene duplication during the evolution or domestication of foxtail millet. In order to validate this hypothesis, we analyzed protein coding sequence diversity among members of the *YUC* genes of foxtail millet. The results of sequence comparison indicated that the *SiYUC13* was part of *SiYUC10*, but no genes with high homology to *SiYUC14* were found. Therefore, we speculated that *SiYUC13* was produced by the partial duplication and insertion of *SiYUC10*.

Furthermore, the genomic colinear analysis of *YUCs* among five grass species revealed potentially functional conservation in *YUC* family members. In particular, *YUC* members (except *SiYUC13* and *SiYUC14*) derived from foxtail millet and green foxtail showed a similar chromosomal distribution, suggesting that foxtail millet was domesticated from green foxtail, as verified in previous studies [48]. *SiYUC13* encoded a protein less than 135 aa at the C-terminal compared with *SiYUC10*, and *SiYUC14* was a unique gene only detected in foxtail millet; these observations implied that these two members might be involved in morphological diversification between foxtail millet and green foxtail.

### 3.2. SiYUC Genes Are Functionally Divergent and Might Be Involved in Hormonal Crosstalk and Stress Response in Foxtail Millet

Previous studies revealed that there was functional redundancy among *YUC* members [18,30]. In this trial, the expressional commonality and variability among *SiYUCs* indicated functional conservation and diversity of *YUC* members in foxtail millet. *SiYUCs* with similar expression profiles might be involved in similar tissue development process [28]. For example, *SiYUC1*, *SiYUC3*, *SiYUC6*, *SiYUC7*, *SiYUC8*, *SiYUC9*, *SiYUC10*, *SiYUC11*, and *SiYUC13* were expressed in developing seeds, implying that they might regulate seed development through conserved function. While the expression of most *SiYUCs* could be detected in seedling leaves, except for *SiYUCs* on chromosome 8 and *SiYUC11* (Figure 5A), expressional correlation analysis between *YUC* members implied that *SiYUC3* and *SiYUC7* might be functionally conservative and redundant in root development regulation.

Functional differentiation among *YUC* genes was also identified through the analysis of expression specificity in this study. The different expression patterns of *YUC* genes imply that they were involved in regulating the development of different tissues and organs in plants [28,29]. For example, the transcription of *SiYUC11* was only detected in pre-flowering panicles, flowering inflorescence secondary branches, and seeds. Moreover, in the first root of the seedling, only *SiYUC2*, *SiYUC3*, *SiYUC6*, *SiYUC7*, *SiYUC10*, and *SiYUC13* transcripts were detectable (Figure 5A), which implied that the expressional diversifications of *SiYUCs* is essential for the functional divergence of *YUC* members in foxtail millet.

The *YUC* genes showed functional conservation in different plants; for example, the overexpression of *AtYUC1* in petunia and rice also resulted in elevated IAA content [17,49,50]. *SiYUC1* (*ZmSPI1*) was mainly expressed in the developing panicle, root, and leaves of germinating seedlings, suggesting that the gene might be involved in lateral organ initiation and development as well as inflorescence development. In maize, *Zmspi1* mutants led to the abnormal initiation of axillary meristematic and lateral organs [35]. *ZmYUC10* is responsible for pollen development and is mainly expressed in tapetal cells, microspores, and mature pollen in maize [47]. Similar results were also obtained for the expression profile of *SiYUC* genes, with *SiYUC2* (a direct homolog of *ZmYUC10*) expressed mainly in the flowering panicle and anthers. *SiYUC6* was mainly expressed in the roots and leaves during the whole life cycle, which might be associated with the development and differentiation of lateral organs; similar results have also been observed in rice *OsYUC6* mutants [49,51]. *SiYUC9/10/13* were mainly expressed in the leaves, panicle, and seeds at the later stages of foxtail millet development, suggesting that these genes might be related to grain yield formation; the same functions were also identified in the rice *Osyuc9* mutant, which exhibit decreased grain weight, increased chalkiness, and slower grain filling rate [37]. *SiYUC11* was specifically expressed in developing seeds and might play a critical role in grain development. *SiYUC11* was homologous to *ZmYUC1* in maize and *OsYUC11* in rice, and it has been reported that the content of IAA in the endosperm of the *Zmyuc1* mutant was significantly decreased and seed weight was reduced by 40% in maize [52]. Mutations in *OsYUC11* also exhibited decreased seed size, increased chalkiness, and reduced grain weight (60–70% of the wild type) in rice [37,53,54]. The haplotype analysis in this trial also showed that *SiYUC11* correlated with the grain weight of the panicle in foxtail millet, which was consistent with the reports from maize and rice.

*Cis*-element analysis indicates that *SiYUCs* was not only involved in the cross-talk of plant hormones, but also the response of foxtail millet to various biotic and abiotic stresses. For instance, methyl jasmonate and salicylic acid response elements were identified in most *SiYUC* genes except *SiYUC5/7/11/13*, implying that these genes were potentially involved in the plant defensive response process. Except for *SiYUC11* and *SiYUC13*, all *SiYUC* genes contained light-responsive elements, which were related to the phototropism response in foxtail millet [55]. The low-temperature responsive elements suggested that *SiYUC* genes might correlate with the IAA-mediated cold response in foxtail millet [56].

### 3.3. Haplotype Variation and Selected Potential of SiYUCs for Yield Improvement of Foxtail Millet

Based on the expression pattern of *SiYUCs* combined with the reported functional study of their homologous genes, superior haplotypes enhancing grain yield of foxtail millet were identified. For example, the dominant haplotypes of *SiYUC2/6/8/11* had longer or thicker panicles or greater panicle grain weight, which might be beneficial for enhancing the yield of foxtail millet. In addition, the proportions of cultivars and landraces carrying different haplotypes were analyzed, which revealed that some advantageous haplotypes that potentially increase the grain yield of foxtail millet have not been effectively utilized in breeding programs. For example, Hap1 and Hap3 of *SiYUC2* were the dominant haplotypes contributing to longer panicles, but have not been selected in cultivars; only Hap2 of *SiYUC2* has been selected during breeding. Hap6 of *SiYUC6* was correlated with thicker panicles and has been selected from landrace to the current cultivar of foxtail millet, although Hap6 is still the minor haplotype in current cultivars, but could be favorably selected in future breeding programs. Moreover, many superior haplotypes, such A Hap1 of *SiYUC8* and Hap1/3 of *SiYUC11,* have been positively selected during foxtail millet breeding, which was correlated with higher panicle grain weight.

Molecular markers utilized for the assisted selection of breeding offspring could be developed by using the SNPs or InDels identified among haplotypes/genotypes in this trial. Zhang et al. used molecular markers to assist in breeding soybeans with a high protein content [57]. Molecular markers were also designed according to the haplotypes of *SiCHL1* to accelerate foxtail millet hybrid breeding [58]. DNA markers were also utilized to identify drought-tolerant genotypes with improved barley yield and drought tolerance [59]. In this trial, Hap3 of *SiYUC2* was the superior haplotype contributing to longer panicles, which could be selected by detecting InDels (i1-i4) in Hap3 to improve foxtail millet breeding efficiency. Hap3 of *SiYUC11* was responsible for higher panicle grain weight, which could be identified by marker development for the detection of InDels (i3 and i4). The findings of this study also imply that the screening and subsequent selection of superior haplotypes could significantly improve the breeding efficiency of foxtail millet in future.

## 4. Materials and Methods

### 4.1. Identification and Phylogenetic Analysis of YUCs in Foxtail Millet, Green Foxtail, and Sorghum

Foxtail millet, green foxtail, and sorghum genomes were retrieved from the Phytozome database (https://phytozome-next.jgi.doe.gov/, accessed date: 27 July 2023), and the YUC protein sequences of maize, rice, and Arabidopsis were downloaded from the maizeGDB (https://www.maizegdb.org/, accessed date: 27 July 2023), Rice-data (https://www.ricedata.cn/, accessed date: 27 July 2023), and TAIR databases (https://www.arabidopsis.org/, accessed date: 27 July 2023), respectively.

The YUC protein sequences of maize, rice, and Arabidopsis were used as query sequences to obtain candidate sequences in the protein databases of foxtail millet, green foxtail, and sorghum, respectively, by using BlastP (default parameter), (E-value < 0.0001). The HMM files of FAD-binding (PF01494), FMO-like motif (PF00743) (the Hidden Markov Model (HMM)) were downloaded from the Pfam database, and the HMM search program was used to search for candidate genes in the protein sequences of foxtail millet, green foxtail, and sorghum, respectively (E-value < 0.001), and the genes containing the above two structural domains were taken as candidate genes. All candidate genes were verified using the CDD and SMART websites, and 14, 12 and 11 members of the *YUC* gene family were obtained. In addition, we performed the full-length sequence comparison of the above reported YUC proteins in Arabidopsis, rice, and maize [24,46,47]. The sequences were aligned by Muscle, with the following parameters: gap open (−2.9), gap extend (0), hydrophobicity multiplier (1.2), max memory in MB (2454), max iterations (8), clustering method (UPGMB), min diag length (24).

MEGA-VI was used to construct a phylogenetic tree of YUC proteins in six plant species. The full-length sequences of 76 YUC proteins (14 OsYUCs, 14 ZmYUCs, 11 AtYUCs, 14 SiYUCs, 12 SvYUCs, 11 SbYUCs) were used to construct an unrooted phylogenetic tree by using the neighbor-joining method with 1000 bootstrap replications, clustering method (UPGMB), model (Poisson model), rates among sites (uniform rates), and gaps data treatment (complete deletion). The gene IDs of the 76 *YUC* genes are listed in Table S2.

### 4.2. Protein Properties and Sequence Analyses of SiYUC Genes

The basic physical and chemical parameters of SiYUC proteins, such as molecular weight, isoelectric point, instability coefficient and hydrophilicity, were analyzed by ProParam (https://web.expasy.org/protparam/, accessed date: 7 August 2023). The prediction and statistics of different secondary structures, such as α-helix, β-folding, extended strand, and irregular coiling, were provided by SOPMA (https://npsa-prabi.ibcp.fr/cgi-bin/npsa_automat.pl?page=/NPSA/npsa_sopma.html, accessed date: 7 August 2023). The subcellular location prediction of SiYUC proteins was performed using PSORT prediction (https://wolfpsort.hgc.jp/, accessed date: 7 August 2023) and softberry (http://www.softberry.com/, accessed date: 7 August 2023). The protein spatial structure prediction was performed by the AlphaFlod Protein Structure Database (https://alphafold.ebi.ac.uk/, accessed date: 29 August 2023).

### 4.3. Cis-Elements Analysis of the SiYUC Gene Promoter

The upstream 2000 bp of the *SiYUC* gene was selected as a promoter for *cis*-element analysis using Plant CARE (http://bioinformatics.psb.ugent.be/webtools/plantcare/html/, accessed date: 7 August 2023) [60].

### 4.4. Chromosomal Distribution and Colinearity Analysis of SiYUCs

The visualization of the chromosomal distribution of the *SiYUCs* and the colinearity analysis of the *YUC* genes in the six species were performed by using TBtools software (v2.001) [61].

### 4.5. Expression Patterns and Subcellular Localization of SiYUCs

The transcriptional data for the *YUC* genes were derived from laboratory data already available, and these tissues were derived from six different growth periods: seedling, three-leaf, nodulation, pregnancy, flowering, and maturity [43]. The gene expression levels were shown by log_2_(FPKM+1) and heatmaps were generated using pheatmap. Correlation analysis and visualization between *YUC* genes expression in each tissue were performed using corrplot software (v0.92) (available at https://github.com/taiyun/corrplot, accessed date: 12 August 2023). Subcellularly localized vector constructs for expression of the fusion protein of YUC and GFP driven by the CaMV (Cauliflower mosaic virus) 35S promoter. The *SiYUC11* and *SiYUC13* coding sequences were obtained by amplification and fused to the transient expression vector PAN580 to construct *Pro35S*∷SiYUC11/13-GFP, which was transformed into foxtail millet protoplasts to investigate the subcellular localization. The primers were used for constructing the expression vectors shown in Table S4.

### 4.6. Haplotype Analysis of the SiYUC Genes and Association with Traits in Foxtail Millet

All SNPs used for haplotype analysis were obtained from resequencing of foxtail millet resource populations [42,43]. Haplotype identification and the analysis of phenotypic association and haplotype network were performed by geneHapR [62].

## 5. Conclusions

We identified 14 *YUC* genes in foxtail millet; these were clustered into four subgroups and colinear with other grass species. All *SiYUCs* showed diversified expression patterns and contained hormonal/light/stress-responsive *cis*-elements. Hap1/3 of *SiYUC2*, Hap6 of *SiYUC6*, Hap1 of *SiYUC8*, and Hap1/3 of *SiYUC11* were the superior haplotypes that should be selected in the breeding process to improve foxtail millet grain yield in future.

## Figures and Tables

**Table 1:** The properties of SiYUC proteins.

Protein ID	No. of aa	MW	Theoretical PI	Instability	GRAVY	Alpha Helix	Extended Strand	Beta Turn	Random Coil	Subcellular Localization
SiYUC1	373	39,976.31	9.68	39.65	−0.006	34.05%	17.16%	8.04%	40.75%	Cytoplasmic
SiYUC2	437	48,793.28	8.44	36.21	−0.181	32.04%	16.48%	6.86%	44.62%	Cytoplasmic
SiYUC3	444	49,407.86	8.22	40.72	−0.141	30.63%	16.89%	7.88%	44.59%	Cytoplasmic
SiYUC4	379	41,632.14	8.67	36.15	−0.026	32.19%	17.68%	7.92%	42.22%	Cytoplasmic
SiYUC5	379	41,460.73	8.68	33.02	−0.020	30.34%	18.47%	8.18%	43.01%	Cytoplasmic
SiYUC6	394	42,950.23	7.08	38.91	0.022	32.23%	18.78%	6.35%	42.64%	Cytoplasmic
SiYUC7	420	46,290.53	8.83	39.42	−0.029	30.48%	19.76%	6.19%	43.57%	Cytoplasmic
SiYUC8	425	45,442.24	9.36	35.90	0.023	33.41%	16.71%	7.76%	42.12%	Cytoplasmic
SiYUC9	400	43,207.52	8.89	45.62	−0.121	34.25%	19.00%	9.00%	37.75%	Cytoplasmic
SiYUC10	395	43,136.18	8.51	48.33	−0.107	32.66%	16.96%	8.10%	42.28%	Cytoplasmic
SiYUC11	385	42,506.07	6.51	32.04	0.029	32.21%	18.44%	6.49%	42.86%	Cytoplasmic
SiYUC12	380	41,522.73	8.68	34.41	−0.029	31.05%	18.95%	8.16%	41.84%	Cytoplasmic
SiYUC13	262	28,541.64	6.46	48.31	0.019	35.11%	14.89%	7.63%	42.37%	Cytoplasmic
SiYUC14	382	41,608.06	8.59	33.80	0.040	31.94%	18.32%	8.12%	41.62%	Cytoplasmic

## Data Availability

The data for this study available from the internet links shown in the paper.

## Supplementary Materials

The following supporting information can be downloaded at: https://www.mdpi.com/article/10.3390/ijms242115637/s1.
