# Supplementary material for: Genome-Wide Characterization and Haplotypic Variation Analysis of the YUC Gene Family in Foxtail Millet (Setaria italica)
Source: Int J Mol Sci. 2023 Oct 27;24(21):15637. doi: 10.3390/ijms242115637 (PMC10648439; doi:10.3390/ijms242115637)
Supplement: Supplementary file 1 [file ijms-24-15637-s001.zip › Figure S1. Identification of conserved motif of YUC proteins in foxtail millet, green foxtail, and sorghum.pdf]

Supplemental Figure:

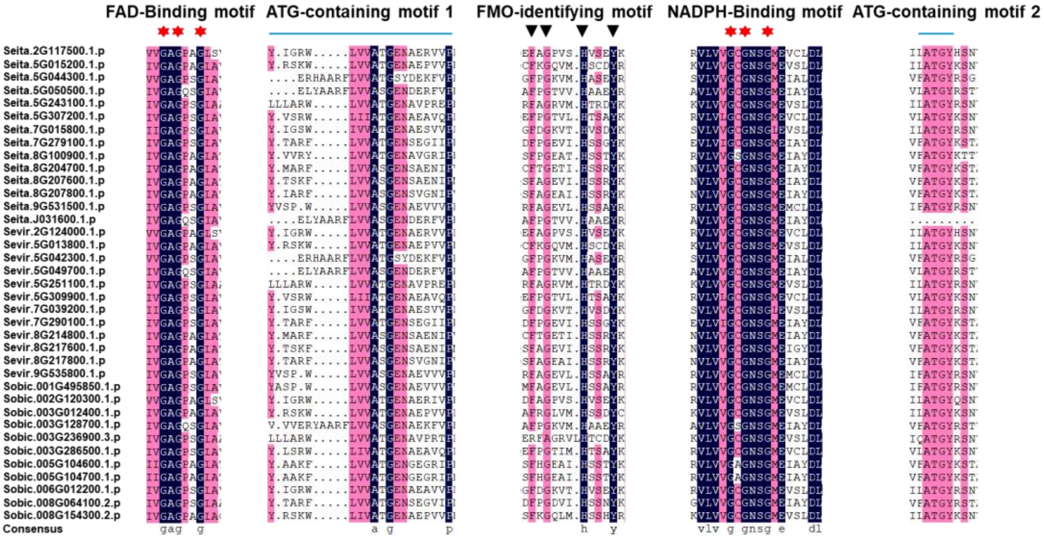

Figure S1. Identification of conserved motif of YUC proteins in foxtail millet, green foxtail, and sorghum. Red hexagonal star, light blue horizontal lines, and black triangles indicate conserved amino acids in the FAD-Binding motif and NADPH-binding motif, ATG-containing motif, and FMO-identifying motif, respectively. Protein IDs used for sequence comparison were listed on the left of the picture. Foxtail millet, green foxtail, sorghum were indicated by Seita, Sevir, Sobic, respectively. Complete protein sequence were used for comparison analysis, and only the typical conserved motifs were shown here.
