# Supplementary material for: Genome-Wide Characterization and Haplotypic Variation Analysis of the YUC Gene Family in Foxtail Millet (Setaria italica)
Source: Int J Mol Sci. 2023 Oct 27;24(21):15637. doi: 10.3390/ijms242115637 (PMC10648439; doi:10.3390/ijms242115637)
Supplement: Supplementary file 1 [file ijms-24-15637-s001.zip › Figure S2. Haplotype analysis of SiYUC6 and SiYUC8. A, E SNPs were used for haplotype analysis of SiYUC6, SiYUC8, respectively.pdf]

Supplemental Figure:

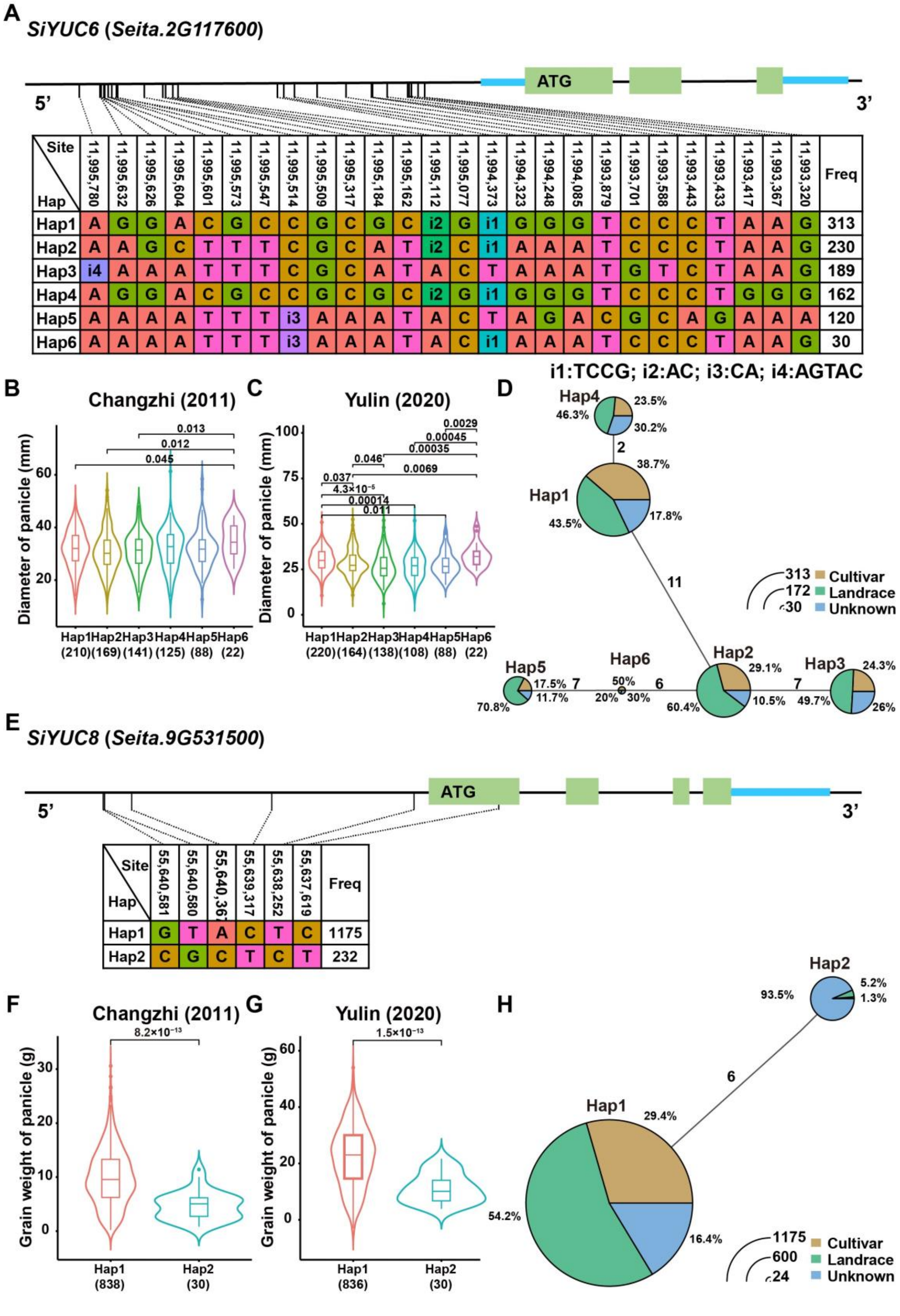

Hap \ Site	11,995,780	11,995,632	11,995,626	11,995,604	11,995,601	11,995,573	11,995,547	11,995,514	11,995,509	11,995,317	11,995,184	11,995,162	11,995,112	11,995,077	11,994,373	11,994,323	11,994,248	11,994,085	11,993,879	11,993,701	11,993,588	11,993,443	11,993,433	11,993,417	11,993,367	11,993,320	Freq
Hap1	A	G	G	A	C	G	C	C	G	C	G	C	i2	G	i1	G	G	G	T	C	C	C	T	A	A	G	313
Hap2	A	A	G	C	T	T	T	C	G	C	A	T	i2	C	i1	A	A	A	T	C	C	C	T	A	A	G	230
Hap3	i4	A	A	A	T	T	T	C	G	C	A	T	A	C	T	A	A	A	T	G	T	C	T	A	A	G	189
Hap4	A	G	G	A	C	G	C	C	G	C	G	C	i2	G	i1	G	G	G	T	C	C	C	T	G	G	G	162
Hap5	A	A	A	A	T	T	T	i3	A	A	A	T	A	C	T	A	G	A	C	G	C	A	G	A	A	A	120
Hap6	A	A	A	A	T	T	T	i3	A	A	A	T	A	C	i1	A	A	A	T	C	C	C	T	A	A	G	30

Hap \ Site	55,640,581	55,640,580	55,640,36	55,639,317	55,638,252	55,637,619	Freq
Hap1	G	T	A	C	T	C	1175
Hap2	C	G	C	T	C	T	232

Figure S2. Haplotype analysis of *SiYUC6* and *SiYUC8*. A, E SNPs were used for haplotype analysis of *SiYUC6*, *SiYUC8*, respectively. light green rectangles indicate exons, straight lines

indicate introns, and SNPs in the promoter and coding sequence of the gene are shown in the upper table. The UTR region is indicated in light blue. Indel was represented by i1-i4. B, C, F, G, The correlation analysis of haplotypes with panicle phenotypes in two different environments. The number in parentheses below the horizontal axis indicates the number of materials contained in the haplotype. D, H Proportion of different foxtail millet resources among haplotypes and haplotype network analysis. Numbers over the line between haplotypes indicate the number of SNPs between them. Percentages indicate the proportion of each foxtail millet resource in that haplotype.
